# Systems-Level Multi-Omics Analysis Resolves the Mechanism of Action of a Novel Multifunctional Nanosystem Against Triple-Negative Breast Cancer

**DOI:** 10.3390/pharmaceutics18080919

**Published:** 2026-07-27

**Authors:** María Pilar Buendia-Nacarino, Dominik Bulfon, Nikola Tom, Mikkel Rohde, Mesut Bilgin, Marja Jäättelä, María Luz Mena, Roberto Alvarez-Fernandez Garcia, Jose L. Luque-Garcia

**Affiliations:** 1 Department of Analytical Chemistry, Faculty of Chemical Sciences, Complutense University of Madrid, 28040 Madrid, Spain; mabuen01@ucm.es (M.P.B.-N.); mariluz@ucm.es (M.L.M.); robalvar@ucm.es (R.A.-F.G.); 2 Cell Death and Metabolism, Center for Autophagy, Recycling and Disease (CARD), Danish Cancer Institute, 2100 Copenhagen, Denmark; dombul@cancer.dk (D.B.); mr@cancer.dk (M.R.);; 3 Lipidomics Core Facility, Danish Cancer Institute, 2100 Copenhagen, Denmark; ntom@cancer.dk (N.T.); mesutb@cancer.dk (M.B.)

**Keywords:** quantitative proteomics, shotgun lipidomics, targeted metabolomics, triple-negative breast cancer, nanotherapeutics

## Abstract

**Background/Objectives:** Triple-negative breast cancer remains a major therapeutic challenge due to its aggressive behavior and limited treatment options. **Methods:** In this study, an integrated multi-omics strategy combining SILAC-based quantitative proteomics, shotgun lipidomics, and targeted metabolomics was applied to characterize the biomolecular response of MDA-MB-231 cells following exposure to the Ag@MSN-Tf-SeNPs nanosystem. **Results and Conclusions:** The complementary analytical platforms provided a comprehensive and system-level view of the cytotoxic effects induced by the nanosystem, highlighting its potential as an antitumoral nanotherapeutic approach. Exposure resulted in reduced cell growth and metastatic potential, associated with DNA damage-induced cell-cycle arrest, disruption of protein biosynthesis, and impaired protein quality control linked to Ca^2+^ homeostasis imbalance. In addition, significant alterations in lipid metabolism were observed, including disruption of cholesterol biosynthesis. These changes, together with disturbances in glycolysis and the tricarboxylic acid cycle, and alterations in NAD^+^/NADH balance, indicate the induction of oxidative stress driven by reactive oxygen species accumulation. Furthermore, the integration of multi-omics data revealed the activation of compensatory mechanisms aimed at restoring cellular homeostasis, including metabolic rewiring and stress-response pathways, although these responses were insufficient to counteract nanosystem-induced cytotoxicity.

## 1. Introduction

Breast cancer remains one of the leading causes of cancer-related mortality among women worldwide [1]. Among its different subtypes, triple-negative breast cancer (TNBC), characterized by the absence of estrogen and progesterone receptors (ER^−^/PR^−^) and human epidermal growth factor receptor 2 (HER2^−^), represents the most aggressive and therapeutically challenging form due to the lack of targeted treatments and poor response to conventional therapies [2].

In this context, nanomedicine has emerged as a promising strategy to overcome the limitations associated with traditional cancer treatments [3], such as systemic toxicity and lack of selectivity [4]. Nanomaterials, typically in the size range of 1–200 nm, offer unique physicochemical properties and can be engineered using a wide variety of materials, including lipids, proteins, and inorganic nanoparticles [5]. Among them, metallic and semi-metallic nanoparticles have attracted increasing attention in oncology due to their potential for controlled drug delivery, targeted action, and intrinsic cytotoxic properties [6,7].

Silver nanoparticles (AgNPs) are among the most extensively studied metallic nanomaterials in cancer therapy [8]. Their cytotoxic activity can be modulated depending on dose and cellular context, enabling selective effects against cancer cells while minimizing toxicity in healthy tissues [9]. AgNPs have been reported to induce apoptosis, cell-cycle arrest, oxidative stress, DNA damage, and disruption of key metabolic pathways [10,11]. Similarly, selenium nanoparticles (SeNPs) have emerged as promising candidates due to their selective anticancer activity [12,13], particularly through the induction of oxidative stress, apoptosis, and cellular senescence in breast cancer models [14].

The incorporation of these nanoparticles into mesoporous silica nanoparticles (MSNs) further enhances their therapeutic potential. MSNs act as versatile nanocarriers, improving stability, reducing off-target toxicity, and enabling controlled delivery of active agents [15,16]. Moreover, the presence of surface hydroxyl groups allows for efficient functionalization for targeted delivery strategies [17,18]. Importantly, the silica shell is not merely an inert support but can substantially influence the biological activity of the nanosystem. Encapsulation of AgNPs within mesoporous silica improves colloidal stability, limits uncontrolled aggregation, modulates the accessibility and release of silver species, and provides a versatile interface for further functionalization, thereby influencing cellular uptake, intracellular trafficking, and overall therapeutic performance [19,20]. Furthermore, surface engineering through the incorporation of targeting ligands or bioactive molecules can modify nanoparticle–cell interactions, protein corona formation, receptor-mediated internalization, and biological selectivity [17,18,19,20].

In our previous work [21], we developed and physicochemically characterized the Ag@MSN-Tf-SeNPs nanosystem and demonstrated its selective antitumoral activity against MDA-MB-231 cells, including reduced cell viability, induction of apoptosis, cell-cycle arrest, and metabolic perturbations identified by untargeted metabolomics. The present nanosystem was therefore designed by exploiting the complementary functions of each component: the AgNP core provides intrinsic cytotoxic activity, the MSN shell enhances stability and enables surface functionalization, transferrin promotes selective uptake through transferrin receptor-mediated endocytosis, and SeNPs further contribute to the antitumoral response. While these findings established the therapeutic potential of the nanosystem, the molecular mechanisms responsible for these biological effects remained largely unresolved.

Therefore, the present study applies an integrated multi-omics workflow [22] combining SILAC-based quantitative proteomics, shotgun lipidomics, and targeted metabolomics to investigate the biomolecular alterations induced by Ag@MSN-Tf-SeNPs in MDA-MB-231 cells. Integrated multi-omics approaches have recently emerged as powerful tools for deciphering nano-biointeractions because they enable the simultaneous characterization of molecular changes occurring at multiple biological levels, providing a more comprehensive understanding of nanoparticle-induced cellular responses than any individual omics technique alone [23,24,25]. This approach enables a system-level characterization of the cellular response, providing deeper insight into the mechanisms of action of the nanosystem and highlighting the potential of multi-omics strategies in nanomedicine research.

## 2. Materials and Methods

### 2.1. Synthesis and Physicochemical Characterization of Ag@MSN-Tf-SeNPs

**Figure 1:**
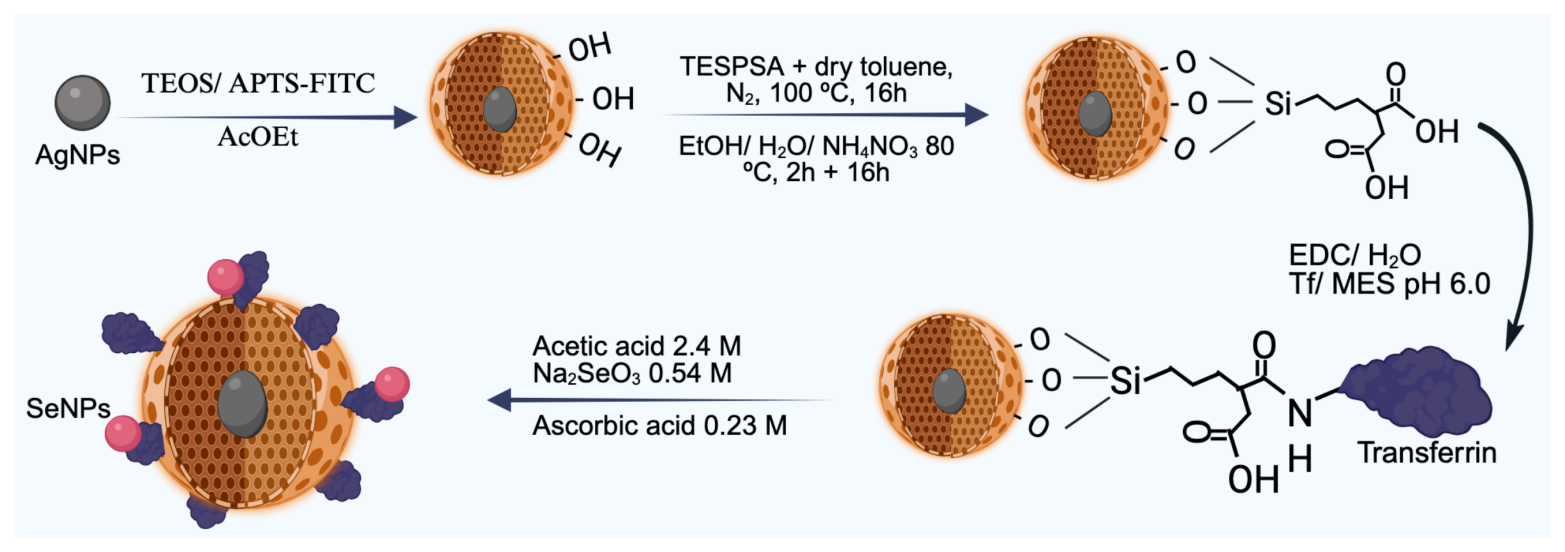
Schematic representation of the synthesis of Ag@MSN-Tf-SeNPs. Created in BioRender. Luque, J. L. (2026) https://BioRender.com/509t54d.

The synthesis of Ag@MSN-Tf-SeNPs has been previously described [21]. Briefly, the nanosystem consisted of a silver nanoparticle core coated with a mesoporous silica shell, using cetyltrimethylammonium bromide (CTAB) as a structure-directing agent for pore formation. Subsequently, the Ag@MSN surface was functionalized with carboxyl groups through grafting of triethoxysilylpropylsuccinic anhydride (TESPSA). Transferrin (Tf) was then covalently attached to the exposed carboxyl groups to promote active targeting toward cancer cells overexpressing transferrin receptors (TfR). In addition, Tf acted as a support for the nucleation of selenium nanoparticles (SeNPs), yielding the final hybrid nanosystem (Figure 1).

To confirm the successful synthesis and physicochemical properties of Ag@MSN-Tf-SeNPs, the obtained nanomaterials were characterized by transmission electron microscopy coupled to energy-dispersive X-ray spectroscopy (JEOL JEM-1400, Tokyo, Japan) (Figure S1 in Supplementary Data). Further physicochemical characterization of the nanosystem has been described elsewhere [21].

### 2.2. Cell Culture

The human triple-negative breast cancer cell line MDA-MB-231 was used as an in vitro model to evaluate the effects of Ag@MSN-Tf-SeNPs on one of the most aggressive and invasive breast cancer subtypes. Cells were maintained in Dulbecco’s Modified Eagle Medium (DMEM) supplemented with 10% (*v*/*v*) fetal bovine serum (FBS) and 1% (*v*/*v*) penicillin/streptomycin under a humidified atmosphere containing 5% CO_2_ at 37 °C.

### 2.3. Quantitative Proteomics

Quantitative proteomic analysis was performed using a stable isotope labeling by amino acids in cell culture (SILAC) strategy. MDA-MB-231 cells were cultured in two different DMEM media: one containing naturally occurring amino acids (“light” medium) and the other supplemented with stable isotope-labeled arginine and lysine (“heavy” medium, ^13^C/^15^N-labeled). Both media were supplemented with 10% (*v*/*v*) dialyzed fetal bovine serum and 100 U/mL penicillin/streptomycin. Culture media were refreshed every 48 h.

After labeling, cells from each condition were divided into untreated controls and cells exposed to 10 mg/L Ag@MSN-Tf-SeNPs for 72 h. Equal amounts of control and treated cells were mixed (1:1) and lysed in buffer containing 50 mM Tris, 150 mM NaCl, 0.2 mM EDTA, and protease inhibitors. Proteins were separated by 10% SDS-PAGE and visualized by Coomassie Blue staining.

Excised gel bands were destained with 25 mM ammonium bicarbonate followed by ammonium bicarbonate/acetonitrile (1:1, *v*/*v*), dehydrated, and rehydrated with trypsin solution (12.5 ng/mL in 25 mM ammonium bicarbonate). In-gel digestion was carried out overnight at 37 °C. Peptides were extracted using sequential extraction with acetonitrile and an aqueous solution containing 5% (*v*/*v*) formic acid. The extracts were then dried and reconstituted in 12 µL of 2% (*v*/*v*) acetonitrile with 0.1% (*v*/*v*) formic acid prior to LC-MS/MS analysis.

Peptide mixtures were first concentrated on a 20 mm × 75 µm C18 pre-column and subsequently separated on a reverse-phase C18 analytical column (500 mm × 75 µm, PepMap RSLC, 2 µm, 100 Å; Thermo Scientific, Waltham, MA, USA) using a linear gradient of 2–40% (*v*/*v*) acetonitrile containing 0.1% (*v*/*v*) formic acid over 240 min at a flow rate of 250 nL/min. Analyses were performed by nanoLC-MS/MS using a Q Exactive HF Orbitrap mass spectrometer (Thermo Scientific) operating in full-scan mode with data-dependent MS/MS acquisition. The fifteen most intense precursor ions with charge states between +2 and +6 were selected for fragmentation using higher-energy collisional dissociation, with a dynamic exclusion time of 27 s.

Raw data were processed using Proteome Discoverer 2.4 (Thermo Scientific) with the MASCOT search engine against the *Homo sapiens* UniProt database (20,416 entries). Variable modifications included methionine oxidation, protein N-terminal acetylation, and SILAC labels on arginine and lysine. Trypsin was selected as the digestion enzyme allowing for up to two missed cleavages. Precursor and fragment mass tolerances were set to 10 ppm and 0.02 Da, respectively. Protein identifications were filtered using the Percolator algorithm with a q-value < 0.01. Relative protein quantification was based on SILAC ratios calculated as the abundance ratio between heavy- and light-labeled peptides. Statistical significance was assessed by Student’s *t*-test, considering *p* < 0.05.

### 2.4. Quantitative Shotgun Lipidomics

For lipidomic analysis, MDA-MB-231 cells were seeded in 6-well plates at a density of 2 × 10^5^ cells/well. After 24 h, cells were exposed in triplicate to 10 mg/L Ag@MSN-Tf-SeNPs for 6, 24, 48, and 72 h, while untreated cells were used as controls. After each exposure time, cells were washed twice with PBS, scraped in PBS, and centrifuged at 500× *g* for 3 min at 4 °C to remove the supernatant.

Lipids were extracted using methyl tert-butyl ether/methanol (MTBE/MeOH, 3:1, *v*/*v*). Briefly, cell pellets were resuspended in 700 µL of extraction solvent containing 25 µL of an internal standard mixture. The 25 µL of internal lipid standards consisted of 100 pmol cholesteryl ester (CE) 15:0-D7, 62.5 pmol Cer 18:1; 2/12:0; 0, 875 pmol free cholesterol (FC)-D4, 62.5 pmol diacylglycerol (DAG) 12:0/12:0, 75 pmol dihexosylceramide (diHexCer) 18:1; 2/17:0; 0, 62.5 pmol HexCer 18:1; 2/12:0; 0, 50 pmol long-chain base (LCB) 17:1; 2, 62.5 pmol lysophosphatidic acid (LPA) 17:0, 50 pmol lysophosphatidylcholine (LPC) 12:0, 50 pmol lysophosphatidylethanolamine (LPE) 13:0, 50 pmol lysophosphatidylglycerol (LPG) 17:1, 62.5 pmol lysophosphatidylinositol (LPI) 13:0, 50 pmol lysophosphatidylserine (LPS) 17:1, 62.5 pmol phosphatidic acid (PA) 12:0/12:0, 62.5 pmol phosphatidylcholine (PC) 12:0/12:0, 75 pmol phosphatidylethanolamine (PE) 12:0/12:0, 50 pmol phosphatidylglycerol (PG) 12:0/12:0, 50 pmol phosphatidylinositol (PI) 8:0/8:0, 50 pmol phosphatidylserine (PS) 12:0/12:0, 50 pmol sphingomyelin (SM) 18:1; 2/12:0; 0, and 50 pmol triacylglycerol (TAG) 17:0/17:0/17:0. Samples were shaken at 1100 rpm for 20 min, followed by addition of 150 µL HPLC-grade water to induce phase separation. After centrifugation at 15,000× *g* for 5 min, the organic and aqueous phases were separated. Both fractions were evaporated to dryness; the aqueous phase was reserved for protein quantification, whereas the organic phase containing lipid extracts was reconstituted in MTBE/MeOH and stored until analysis at −80 °C.

Mass spectrometric analysis was performed in positive and negative ion modes on an Orbitrap Fusion™ mass spectrometer (Thermo Fisher Scientific, Waltham, MA, USA) coupled to TriVersa NanoMate (Advion Biosciences, Ithaca, NY, USA) for automated and direct nanoESI infusion. The mass spectrometric settings were adapted from our previously published method with minor modifications [26]. Samples analyzed in positive ionization mode were infused using a back pressure of 1.25 psi and an ionization voltage of 0.95 kV, whereas in negative ionization mode, a back pressure of 0.7 psi and an ionization voltage of −1.06 kV were applied. Data acquisition consisted of MS and MS/MS scans in both ionization modes. The software LipidXplorer (version 1.2.7) was used for reporting the identified lipid species with their detected *m*/*z* and the corresponding intensities for the associated precursor and fragment ions acquired in MS and MS/MS data. Absolute molar quantities and relative molar quantities (mol%) were calculated using an in-house built software LipidMetrix (publication in preparation). Absolute quantities were normalized to mg protein and are expressed as pmol/μg protein.

### 2.5. Targeted Metabolomics Analysis

Targeted metabolomics was performed to evaluate alterations in key metabolites related to cellular bioenergetics, the tricarboxylic acid (TCA) cycle, and folate metabolism after exposure to Ag@MSN-Tf-SeNPs. MDA-MB-231 cells were seeded in P100 culture dishes and exposed to 10 mg/L of the nanosystem for 72 h. After treatment, cells were washed with 0.9% (*m*/*v*) NaCl solution, and intracellular metabolites were extracted by addition of 100 µL methanol pre-cooled at −20 °C followed by cell scraping. Subsequently, 400 µL of ice-cold 0.4% (*v*/*v*) formic acid was added, and extracts were transferred to Eppendorf tubes for further processing.

Metabolite quantification was carried out using LC-QqQ-MS on a Shimadzu LC/MS-8030 system operating in multiple reaction monitoring (MRM) mode. External calibration curves were prepared for each analyte in 20% (*v*/*v*) methanol in water.

For bioenergetic profiling, the following metabolites were quantified: adenosine triphosphate (ATP), adenosine diphosphate (ADP), reduced nicotinamide adenine dinucleotide (NADH), oxidized nicotinamide adenine dinucleotide (NAD^+^), reduced nicotinamide adenine dinucleotide phosphate (NADPH), and oxidized nicotinamide adenine dinucleotide phosphate (NADP^+^). Chromatographic separation was performed using a Poroshell 120 Phenyl-Hexyl column (2.1 × 50 mm, 2.7 µm; Agilent, Santa Clara, CA, USA). Mobile phase A consisted of water/methanol (97:3, *v*/*v*) containing 10 mM tributylamine and 3 mM acetic acid, whereas mobile phase B was methanol. Gradient elution was applied over a total run time of 11 min at a flow rate of 0.30 mL/min with an injection volume of 20 µL.

To assess additional metabolic pathways affected by treatment, selected metabolites involved in the TCA cycle and folate metabolism were also quantified, including folic acid, citric acid, α-ketoglutaric acid, succinic acid, fumaric acid, uric acid, and malic acid. Analyses were performed using a Gemini C18 column (150 × 2 mm, 5 µm, 110 Å; Phenomenex, Torrance, CA, USA). Mobile phase A was water containing 0.1% (*v*/*v*) formic acid and mobile phase B was acetonitrile. The chromatographic run time was 10 min at a flow rate of 0.40 mL/min with an injection volume of 10 µL.

For all analyses, nitrogen was used as nebulizing and drying gas at flow rates of 1.5 and 15.0 mL/min, respectively. The ionization voltage was set at 4.5 kV, the desolvation line temperature at 250 °C, and detector voltage at 1.8 kV. Quantifier transitions for each metabolite were selected according to the highest signal intensity under optimized collision-induced dissociation conditions.

### 2.6. Senescence Assay

Cellular senescence was evaluated by detection of senescence-associated β-galactosidase (SA-β-gal) activity using a cytochemical staining kit (Sigma-Aldrich, St. Louis, MO, USA). MDA-MB-231 cells were exposed to 10 mg/L Ag@MSN-Tf-SeNPs for 72 h under standard culture conditions (37 °C, 5% CO_2_). In parallel, two positive controls were included: a senescence control treated with 10 µM etoposide and an apoptosis-related cytotoxicity control treated with 50 µM etoposide for the same exposure time.

After treatment, cells were washed with PBS and fixed for 7 min at room temperature using a fixation solution containing 20% (*v*/*v*) formaldehyde, 2% (*v*/*v*) glutaraldehyde, 70.4 mM Na_2_HPO_4_, 14.7 mM KH_2_PO_4_, 1.37 M NaCl, and 26.8 mM KCl. Cells were then washed again with PBS and incubated with the β-galactosidase staining solution according to the manufacturer’s instructions. Following overnight incubation at 37 °C in the absence of CO_2_, stained cells were examined and imaged using an EVOS XL Core inverted phase-contrast microscope (Thermo Fisher Scientific). β-galactosidase staining was quantified using ImageJ software (version 1.54t).

### 2.7. Galectin-3 Puncta Assay

Lysosomal membrane damage was assessed through galectin-3 (Gal3) puncta formation using an immunofluorescence-based assay essentially as described previously [27]. MDA-MB-231 cells were seeded in 96-well plates at a density of 3000 cells/well. After 24 h, cells were exposed to 10 mg/L Ag@MSN-Tf-SeNPs for 72 h. Following treatment, cells were fixed with 100 µL of 4% (*v*/*v*) paraformaldehyde for 10 min at room temperature, washed twice with PBS, and permeabilized with 50 µL of cold methanol for 10 min. After two additional PBS washes, cells were blocked with 40 µL blocking buffer supplemented with 5% (*v*/*v*) goat serum for 15 min. Cells were then incubated for 2 h with primary antibodies diluted in blocking buffer containing 5% (*v*/*v*) goat serum: anti-galectin-3 (rat, 1:200) and anti-α-tubulin (mouse, 1:200) (Invitrogen (Carlsbad, CA, USA)), Thermo Fisher Scientific). Subsequently, cells were washed three times with PBST (PBS containing 0.1% Tween-20 (Molecular Devices, San Jose, CA, USA)) for 10 min each and incubated for 30 min with fluorophore-conjugated secondary antibodies anti-rat and anti-mouse (Invitrogen, Thermo Fisher Scientific (Waltham, MA, USA)), emitting in the red and far-red channels, respectively. After three additional PBST washes, nuclei were stained with Hoechst solution (1:1000 in PBS) for 3 min, followed by a final PBS wash. Images were acquired using an ImageXpress Confocal HT microscope (San Jose, CA, USA) equipped with 40× immersion objectives and filter sets for DAPI, FITC, Texas Red, and Cy5. Nine images were collected per well. Image acquisition and quantitative analysis were performed using MetaXpress software.

## 3. Results

### 3.1. SILAC-Based Quantitative Proteomics Reveals Molecular Targets Altered After Ag@MSN-Tf-SeNPs Exposure

To gain deeper insight into the molecular response induced by Ag@MSN-Tf-SeNPs in triple-negative breast cancer cells, a SILAC-based quantitative proteomics approach was performed in MDA-MB-231 cells exposed to 10 mg/L of the nanosystem for 72 h.

The proteomic workflow enabled the identification of a total of 1169 proteins. Among them, 70 proteins showed statistically significant changes in abundance (*p* < 0.05), including 29 downregulated and 41 upregulated proteins (Table 1). All identified proteins were taxonomically assigned to *Homo sapiens* (UniProt proteome UP000005640).

Differentially expressed proteins were mainly associated with cellular stress response, protein folding and quality control, metabolic regulation, cytoskeleton organization, and organelle homeostasis. Several altered proteins were related to key intracellular compartments previously implicated in nanosystem-mediated cytotoxicity, including the endoplasmic reticulum, mitochondria, and nucleus.

Overall, these results indicate that Ag@MSN-Tf-SeNPs exposure induces a coordinated proteomic response involving both damage-associated pathways and adaptive cellular mechanisms.

### 3.2. Quantitative Shotgun Lipidomics Complements the Characterization of Biomolecular Alterations Induced by Ag@MSN-Tf-SeNPs Exposure

**Figure 2:**
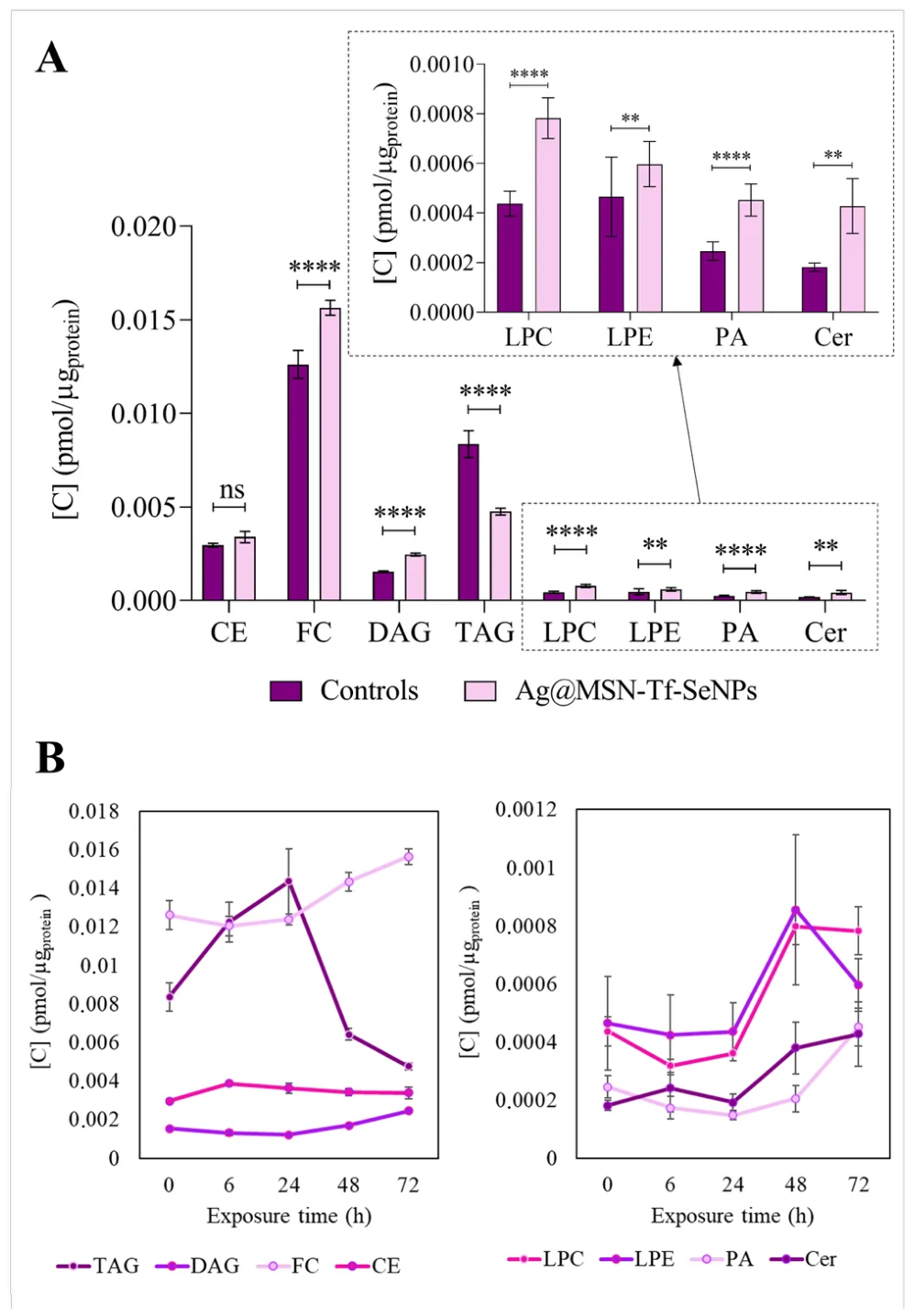
Most significantly altered lipid classes. (**A**) Comparison between untreated MDA-MB-231 control cells and cells exposed to Ag@MSN-Tf-SeNPs for 72 h. Data were analyzed by one-way ANOVA followed by Bonferroni’s multiple comparisons test: **** *p* < 0.0001, ** *p* < 0.01, ns, not significant. (**B**) Time-dependent variation in lipid levels after 6, 24, 48, and 72 h of exposure.

To further investigate the molecular pathways affected in MDA-MB-231 cells after treatment with Ag@MSN-Tf-SeNPs, quantitative shotgun lipidomics was performed. Lipid profiles were compared between untreated control cells and cells exposed to the nanosystem for 72 h (Figure 2A). In addition, time-dependent lipid alterations were monitored after 6, 24, 48, and 72 h of exposure (Figure 2B) to monitor the variation in lipid classes at key exposure times.

The analysis included the main cellular lipid classes: CE, FC, CL, LPA, LPC, LPE, LPG, LPI, LPS, PA, PC, PE, PG, PI, PS, Cer, DAG, and TAG. Quantitative data for all lipid classes are provided in Table S1 in the Supplementary Materials.

Lipidomics analysis revealed significant increases in FC, DAG, LPC, LPE, PA, and Cer levels after nanosystem exposure. In contrast, TAG levels significantly decreased, whereas CE levels remained unchanged.

Time-course analysis showed that FC and Cer reached their highest levels after 72 h of exposure, while PA, LPC, LPE exhibited their maxima at 48 h. In contrast, TAG reached its highest level at 24 h, showing a significant decrease at 48 h. CE and DAG did not show a remarkable change during the time course.

### 3.3. Targeted LC–MS/MS Metabolomics Reveals Alterations in Bioenergetic and Central Metabolic Pathways

**Figure 3:**
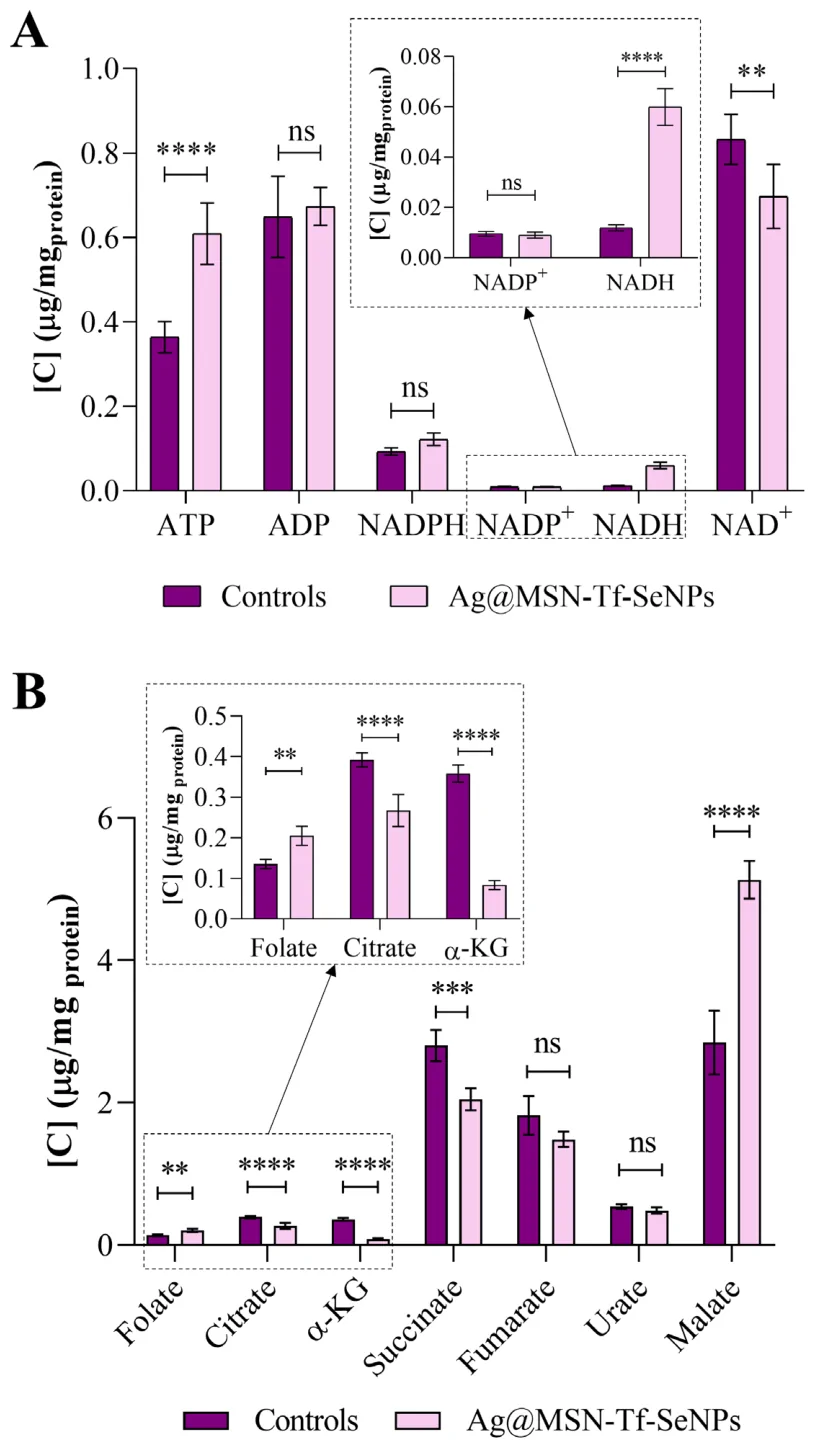
Quantitative analysis of key metabolites involved in (**A**) cellular energy metabolism and (**B**) folate and tricarboxylic acid (TCA) cycle pathways in MDA-MB-231 cells. Comparison between untreated control cells and cells exposed to Ag@MSN-Tf-SeNPs for 72 h. Data were analyzed by one-way ANOVA followed by Bonferroni’s multiple comparisons test: **** *p* < 0.0001, *** *p* < 0.001, ** *p* < 0.01, ns, not significant.

Intracellular levels of key bioenergetic metabolites, including ATP, ADP, NAD^+^, NADH, NADP^+^, and NADPH, were quantified in MDA-MB-231 cells after exposure to 10 mg/L Ag@MSN-Tf-SeNPs (Figure 3A). Cellular extracts were analyzed by LC-QqQ-MS operating in multiple reaction monitoring (MRM) mode to ensure high sensitivity and selectivity. Metabolite concentrations were normalized to total protein content determined by the Bradford assay. Statistical analysis was performed using one-way ANOVA (95% confidence level) followed by Bonferroni’s post hoc test. The results showed a significant increase in ATP and NADH levels, whereas NAD^+^ levels were decreased in treated cells compared to controls.

In addition, selected metabolites involved in the tricarboxylic acid (TCA) cycle and one-carbon metabolism were quantified due to their relevance in cellular metabolic regulation (Figure 3B). A decrease in citrate, α-ketoglutarate, and succinate levels was observed after nanosystem exposure. In contrast, malate levels were increased. Furthermore, folate levels, associated with one-carbon metabolism, were also found to be elevated.

### 3.4. Senescence-Associated β-Galactosidase Activity Induced by Ag@MSN-Tf-SeNPs

Previous results demonstrated that exposure of MDA-MB-231 cells to Ag@MSN-Tf-SeNPs induced cell-cycle arrest. To further evaluate whether this effect was associated with the induction of cellular senescence, senescence-associated β-galactosidase (SA-β-gal) activity was assessed as a senescence biomarker.

**Figure 4:**
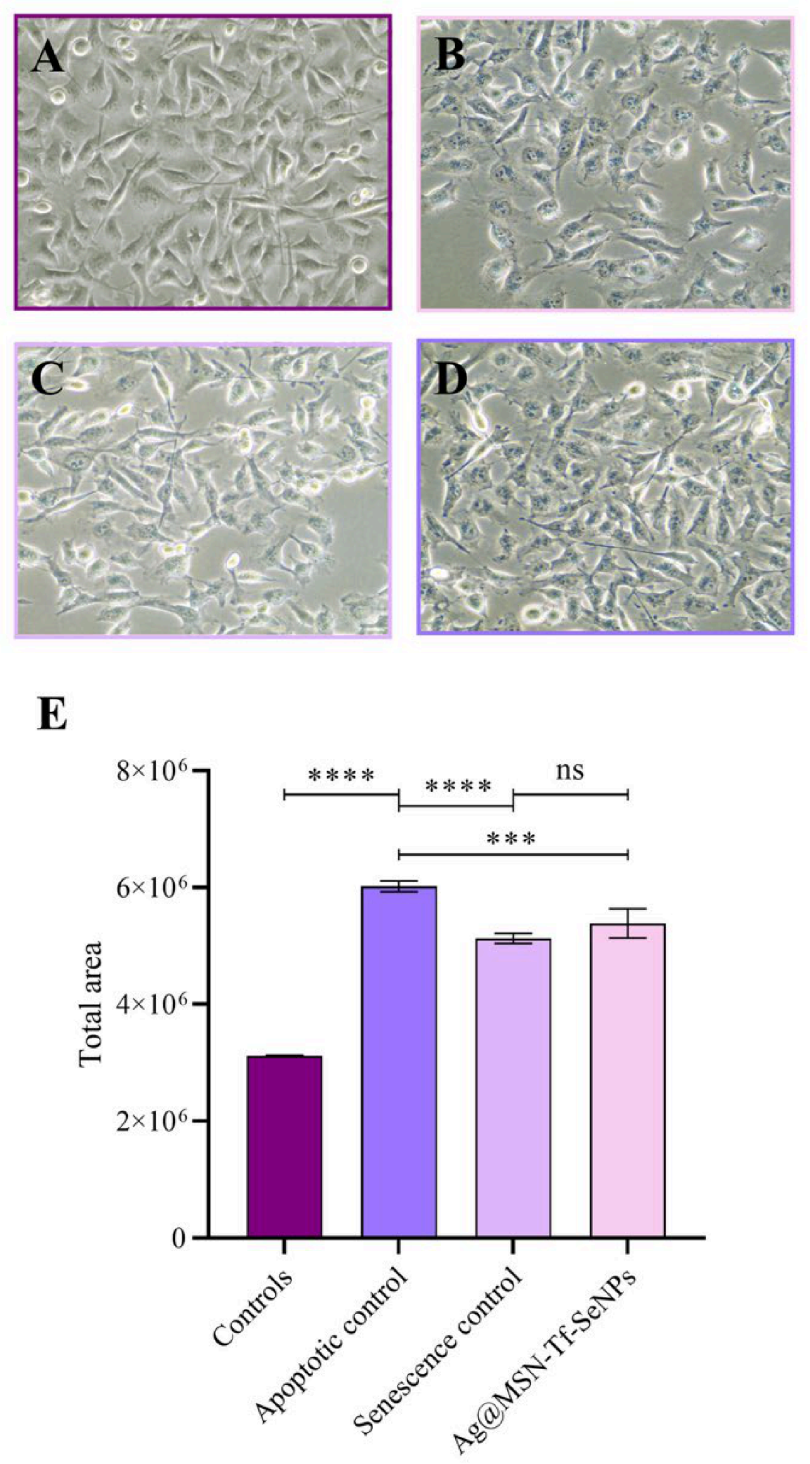
Senescence-associated β-galactosidase (SA-β-gal) staining in MDA-MB-231 cells. Blue staining indicates β-galactosidase activity. (**A**) Untreated control cells; (**B**) cells exposed to Ag@MSN-Tf-SeNPs; (**C**) cells treated with 10 µM etoposide (senescence-positive control); (**D**) cells treated with 50 µM etoposide (apoptosis-related cytotoxicity control); (**E**) Quantification of SA-β-gal staining using ImageJ software. Data were analyzed by one-way ANOVA followed by Bonferroni’s multiple comparisons test: **** *p* < 0.0001, *** *p* < 0.001, ns, not significant.

Control cells showed low levels of β-galactosidase staining, consistent with basal senescence levels (Figure 4A). In contrast, cells exposed to Ag@MSN-Tf-SeNPs for 72 h exhibited an increased number of blue-stained cells, indicating enhanced SA-β-gal activity (Figure 4B). For comparison, cells treated with 10 µM etoposide (senescence-positive control) (Figure 4C) and 50 µM etoposide (apoptosis-related cytotoxicity control) (Figure 4D) were also analyzed. Ag@MSN-Tf-SeNPs-treated cells displayed staining levels higher than untreated controls and comparable to those observed in the senescence-positive control, although lower than those observed under the apoptosis-inducing condition. Semi-quantitative results were obtained from the images using ImageJ software (Figure 4E).

### 3.5. Galectin-3 Puncta Formation Indicates Lysosomal Damage Induced by Ag@MSN-Tf-SeNPs

**Figure 5:**
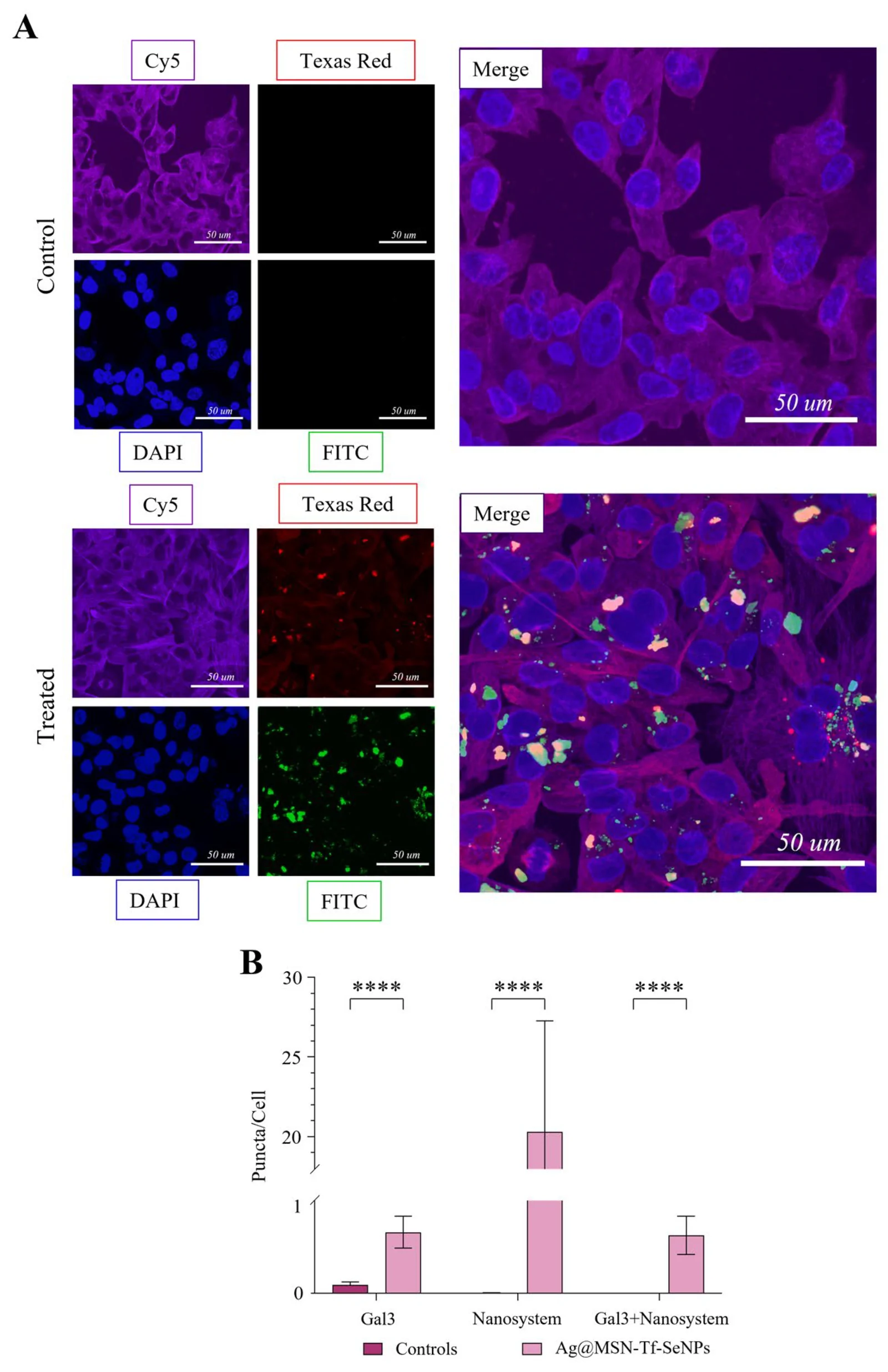
(**A**) Representative confocal images of MDA-MB-231 control cells and Ag@MSN-Tf-SeNPs treated cells, stained with Cy5 for tubulin, DAPI for nucleus, Texas Red for gal 3 puncta and FITC in the mesoporous silica nanoparticle. (**B**) Number of red puncta (Gal 3) and green spots (nanosystems) per number of cells in each well. Data were analyzed by ANOVA with Bonferroni’s multiple comparisons test: **** *p* < 0.0001.

To evaluate lysosomal membrane damage induced by Ag@MSN-Tf-SeNPs, galectin-3 (Gal3) puncta formation was analyzed in MDA-MB-231 cells after 72 h of exposure. Control and treated cells (n = 4 per condition) were compared using fluorescence microscopy (Figure 5A).

Control cells did not show detectable Gal3 puncta, consistent with the absence of lysosomal damage. In contrast, treated cells exhibited distinct Gal3 puncta stained in the Texas Red channel, together with green fluorescent signals corresponding to Ag@MSN-Tf-SeNPs. Merged images enabled the simultaneous visualization of cytoskeletal staining (tubulin), nuclei, and Gal3 puncta, revealing colocalization between Gal3-positive structures and nanosystem-associated fluorescence.

Quantitative analysis (Figure 5B) showed a significant increase in the number of Gal3 puncta per cell in treated samples compared to controls. Notably, the majority of Gal3 puncta detected in treated cells were spatially associated with nanosystem-derived fluorescence signals.

## 4. Discussion

To gain deeper insight into the biomolecular mechanisms underlying the antitumoral activity of Ag@MSN-Tf-SeNPs in triple-negative breast cancer (TNBC), an integrated multi-omics approach combining SILAC-based quantitative proteomics, shotgun lipidomics, and targeted metabolomics was applied. Our previous study [21] established the synthesis and physicochemical characterization of the Ag@MSN-Tf-SeNPs nanosystem and demonstrated its antitumoral activity in MDA-MB-231 cells, including apoptosis induction, cell-cycle arrest, and metabolic alterations identified by untargeted metabolomics. Building upon these findings, the present work aimed to elucidate the molecular mechanisms underlying these phenotypic effects through the integration of quantitative proteomics, shotgun lipidomics, and targeted metabolomics.

**Figure 6:**
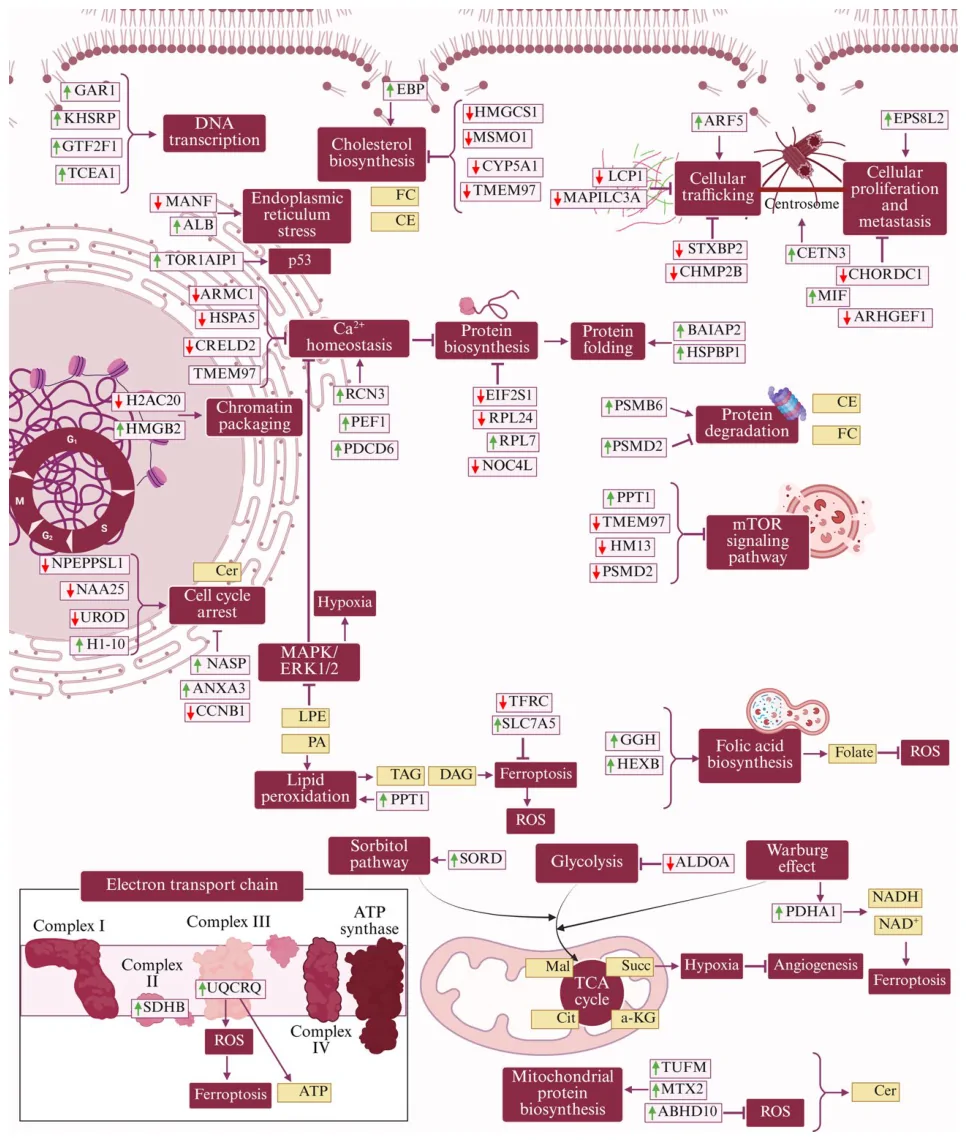
Summary of the major biomolecular pathways disrupted by Ag@MSN-Tf-SeNPs exposure in MDA-MB-231 cells, integrating alterations in proteins, lipids, and metabolites. Created in BioRender. Luque, J. L. (2026) https://BioRender.com/ck560na.

Overall, the results reveal a coordinated disruption of multiple interconnected cellular processes, including nuclear integrity, endoplasmic reticulum (ER) function, mitochondrial metabolism, lipid homeostasis, lysosomal integrity, and intracellular trafficking (Figure 6). Importantly, these alterations are accompanied by the activation of compensatory mechanisms aimed at maintaining cellular survival, highlighting a dynamic balance between damage induction and adaptive responses.

At the nuclear level, several proteins involved in chromatin organization and DNA integrity were significantly altered. The downregulation of histone H2AC20 and HMGB2 suggests impaired nucleosome stability and DNA structural organization, potentially increasing genomic instability. In parallel, proteins such as NPEPPSL1, NAA25, UROD, and H1-10 were upregulated, indicating an attempt to compensate for DNA damage and maintain chromatin organization [28,29,30,31,32]. These alterations are consistent with the observed downregulation of CCNB1, a key regulator of the G2/M transition, supporting the induction of cell-cycle arrest [33]. This effect is further reinforced by the accumulation of ceramides (Cer) (Figure 2A), as evidenced by lipidomic analysis, which are known to promote G0/G1 arrest and cellular senescence (Figure 4) through stress signaling pathways [34].

Interestingly, the overexpression of NASP and annexin-related pathways (ANXA3) suggests activation of survival mechanisms aimed at restoring proliferation capacity and promoting resistance to cellular stress [35]. In addition, the upregulation of transcription-related proteins (GAR1, KHSRP, GTF2F1, TCEA1) indicates increased transcriptional activity, likely as a compensatory response to sustain protein synthesis under stress conditions [36,37,38]. At the same time, the downregulation of EIF2S1 and RPL24 suggests impaired translation efficiency, indicating a decoupling between transcription and translation processes under stress conditions [39].

The ER emerges as a central hub of disruption [40]. The downregulation of key chaperones such as HSPA5, CRELD2, and TMEM97 indicates impaired protein folding capacity and disruption of Ca^2+^ homeostasis, leading to ER stress and activation of apoptosis pathways [41,42,43]. At the same time, compensatory upregulation of RCN3, PEF1, and PDCD6 suggests activation of calcium-buffering mechanisms to restore ER function [44,45]. This dual response highlights the coexistence of damage and adaptive signaling. Proteostasis is further compromised by alterations in the ubiquitin–proteasome system. While PSMB6 upregulation suggests increased degradation of damaged proteins, the downregulation of PSMD2 indicates impaired 26S proteasome function, potentially leading to accumulation of misfolded proteins and reinforcing ER stress [21,46].

One of the most prominent alterations observed was the disruption of lipid metabolism, particularly cholesterol biosynthesis. Proteomic data showed downregulation of key enzymes involved in sterol biosynthesis, including HMGCS1, MSMO1, and CYP51A1 [47,48,49]. This is consistent with lipidomic results showing increased free cholesterol (FC), unchanged cholesteryl esters (CE), increased DAG and Cer levels, and decreased TAG levels at later time points (Figure 2). These findings indicate impaired cholesterol esterification and altered lipid storage, affecting membrane composition and signaling.

The overexpression of EBP suggests activation of compensatory sterol biosynthesis pathways [50], although insufficient to restore lipid homeostasis. In addition, TMEM97 downregulation may contribute to altered cholesterol trafficking and homeostasis [51]. Importantly, the combination of altered cholesterol metabolism and lysosomal damage (as demonstrated by Gal3 puncta formation) suggests dysfunction of the lysosome–mTORC1 axis [52].

Galectin-3 puncta formation demonstrated lysosomal membrane damage induced by Ag@MSN-Tf-SeNPs (Figure 5), indicating loss of lysosomal integrity. The colocalization of Gal3 puncta with nanosystem fluorescence strongly supports a direct interaction between the nanosystem and lysosomal membranes. Proteomic alterations in STXBP2 and CHMP2B further support disruption of vesicular trafficking and membrane repair [53,54]. In parallel, MAP1LC3A downregulation suggests impaired autophagy, indicating a blockade of the autophagy–lysosome axis [55].

Cytoskeletal organization was also affected, as indicated by the downregulation of LCP1, ARHGEF1, and CHORDC1, impairing actin dynamics, cellular motility, and RhoA signaling pathways [56,57,58]. Conversely, the overexpression of BAIAP2, HSPBP1, and ARF5 suggests activation of compensatory mechanisms aimed at restoring cytoskeletal integrity and intracellular trafficking [59,60,61], while alterations in centrosome-associated proteins further indicate disruption of mitotic regulation [62].

Mitochondrial metabolism was profoundly affected. The downregulation of ALDOA suggests impaired glycolysis, while PDHA1 upregulation indicates enhanced conversion of pyruvate into acetyl-CoA [63]. Targeted metabolomics revealed decreased levels of citrate, α-ketoglutarate, and succinate, indicating disruption of the TCA cycle. Reduced succinate levels may promote degradation of HIF-1α, impairing hypoxia adaptation [64]. In contrast, increased malate levels may represent a compensatory response, although insufficient due to the observed NAD^+^ depletion and NADH accumulation, indicating redox imbalance (Figure 3) [65]. Interestingly, the increase in ATP levels together with the altered ATP/ADP ratio suggests the activation of compensatory energetic pathways to sustain cellular bioenergetics under metabolic stress conditions. In addition, the combination of elevated NADH levels, reduced TCA intermediates, and progressive depletion of TAG levels may indicate enhanced fatty acid utilization as an alternative energy source. This metabolic adaptation could be associated with increased lipid catabolism and β-oxidation-related processes contributing to NADH production under Ag@MSN-Tf-SeNPs exposure.

Mitochondrial dysfunction is closely linked to increased ROS production. Upregulation of SDHB and UQCRQ suggests enhanced electron leakage from the electron transport chain [66]. In parallel, the lipidomic profile showed increased PA, LPE, Cer and DAG levels, together with a marked decrease in TAG levels at later exposure times, indicating pronounced lipid remodeling and altered lipid storage under Ag@MSN-Tf-SeNPs exposure. These changes, combined with NAD^+^ depletion and NADH accumulation, may be compatible with enhanced lipid catabolism and oxidative stress. However, direct evidence of lipid peroxidation was not obtained in this study, since oxidized phospholipids or PUFA-containing lipid species were not specifically quantified. Therefore, ferroptosis should be considered a plausible mechanism suggested by the integrated redox and lipidomic alterations, but requiring further validation [67]. In this context, the upregulation of PPT1, a lysosomal thioesterase, may reflect altered lysosomal function and protein depalmitoylation processes rather than direct lipid remodeling. This interpretation is consistent with the Gal3 puncta assay, which demonstrated lysosomal membrane damage after nanosystem exposure.

Cancer cells appear to activate alternative metabolic pathways to compensate for TCA disruption. The upregulation of SORD suggests activation of the sorbitol pathway, contributing to NAD^+^ depletion [68] (Figure 3A), while PDHA1 upregulation may support metabolic rewiring consistent with the Warburg effect [64]. However, these compensatory mechanisms are insufficient to restore metabolic balance.

Adaptive mitochondrial responses were also observed, including upregulation of TUFM, MTX2, and ABHD10, supporting mitochondrial protein synthesis, import, and redox homeostasis [68,69,70]. However, increased ceramide levels and ROS accumulation likely compromise mitochondrial membrane integrity and function.

Finally, alterations in ARMC1 and previous observations of membrane damage [70] suggest impairment of mitochondrial and cellular membrane integrity, affecting Ca^2+^ homeostasis, energy production, and intracellular signaling.

Taken together, the combined proteomic, lipidomic, and metabolomic data support a model in which Ag@MSN-Tf-SeNPs induce a multi-level disruption of cellular homeostasis. The nanosystem triggers ER stress, lipid metabolism dysregulation, lysosomal damage, mitochondrial dysfunction, and redox imbalance, ultimately promoting cell death through apoptosis and possibly involving ferroptosis-related mechanisms (Figure 6). Importantly, this study demonstrates that the integration of complementary analytical platforms enables a system-level understanding of nanosystem-induced cytotoxicity, highlighting the value of multi-omics approaches in the characterization of complex biological responses.

A limitation of the present study is that the integrated multi-omics analyses were performed in a single TNBC cell line (MDA-MB-231). Although this cell line is a well-established model for aggressive triple-negative breast cancer and has been extensively used in nanomedicine research, TNBC comprises a heterogeneous group of tumors with distinct molecular and metabolic characteristics. Therefore, additional studies using complementary TNBC models and, ultimately, in vivo systems will be necessary to determine the extent to which the molecular mechanisms identified here are conserved across different TNBC subtypes and to further validate the therapeutic potential of the Ag@MSN-Tf-SeNPs nanosystem.

## 5. Conclusions

This study demonstrates that the integration of complementary analytical platforms, including SILAC-based quantitative proteomics, shotgun lipidomics, and targeted metabolomics, provides a comprehensive and robust framework for elucidating the molecular mechanisms underlying nanosystem-induced cytotoxicity.

The multi-omics approach revealed a coordinated disruption of key biomolecular pathways in MDA-MB-231 cells exposed to Ag@MSN-Tf-SeNPs, including DNA damage responses, protein homeostasis imbalance, lipid metabolism dysregulation, mitochondrial dysfunction, and redox alterations. In particular, the combined datasets support the occurrence of lipid remodeling and redox imbalance, impairment of the tricarboxylic acid (TCA) cycle, and alterations in cellular energetic metabolism, highlighting the interplay between metabolic stress and cell death mechanisms.

Importantly, the integration of proteomic, lipidomic, and metabolomic data enabled the identification of interconnected pathways involving endoplasmic reticulum stress, lysosomal dysfunction, and mitochondrial impairment, providing a system-level understanding of the cellular response to the nanosystem.

Overall, these findings highlight the potential of Ag@MSN-Tf-SeNPs as an effective antitumoral strategy against triple-negative breast cancer and, more importantly, demonstrate the value of multi-omics analytical approaches for the comprehensive characterization of complex biological responses in nanomedicine.

## Figures and Tables

**Table 1:** Statistically significant altered proteins identified by SILAC-based quantitative proteomics (*p* < 0.05; RSD < 30%) in MDA-MB-231 cells exposed to Ag@MSN-Tf-SeNPs compared with untreated control cells.

Common Name	Accession	Protein Name	SILAC Ratio (Log)	RSD	Mascot Score
CRELD2	Q6UXH1	Protein disulfide isomerase CRELD2	−0.73	0	31
KRAS	P01116	GTPase Kras	−0.68	0	43
AIMP1	Q12904	Aminoacyl tRNA synthase complex-interacting multifunctional protein 1	−0.64	0	52
EHD2	Q9NZN4	EH domain-containing protein 2	−0.52	0	30
ARMC1	Q9NVT9	Armadillo repeat-containing protein 1	−0.52	9.61	20
STXBP2	Q15833	Syntaxin-binding protein 2	−0.46	0	30
CCNB1	P14635	G2/mitotic-specific cyclin-B1	−0.43	26.54	36
NAA25	Q14CX7	N-alpha-acetyltransferase 25, NatB auxiliary subunit	−0.41	0	28
HMGCS1	Q01581	Hydroxymethylglutaryl-CoA synthase, cytoplasmic	−0.38	12.6	21
NOC4L	Q9BVI4	Nucleolar complex protein 4 homolog	−0.34	0	18
CYP51A1	Q16850	Lanosterol 14-alpha demethylase	−0.34	17.48	90
ARHGEF1	Q92888	Rho guanine nucleotide exchange factor 1	−0.33	27.74	20
LCP1	P13796	Plastin-2	−0.29	24.17	1080
MAP1LC3A	Q9H492	Microtubule-associated proteins 1A/1B light chain 3A	−0.29	9.2	86
MSMO1	Q15800	Methylsterol monooxygenase 1	−0.29	21.8	107
UROD	P06132	Uroporphyrinogen decarboxylase	−0.28	28.87	146
CHMP2B	Q9UQN3	Charged multivesicular body protein 2b	−0.27	0.47	16
NPEPPSL1	A6NEC2	Puromycin-sensitive aminopeptidase-like protein	−0.27	0	18
EIF2S1	P05198	Eukaryotic translation initiation factor 2 subunit 1	−0.26	13.51	68
MANF	P55145	Mesencephalic astrocyte-derived neurotrophic factor	−0.24	3.4	244
HSPA5	P11021	Endoplasmic reticulum chaperone BiP	−0.24	22.38	989
TFRC	P02786	Transferrin receptor protein 1	−0.22	23	25
HM13	Q8TCT9	Minor histocompatibility antigen H13	−0.21	21.27	243
TMEM97	Q5BJF2	Sigma intracellular receptor 2	−0.19	4.19	52
ALDOA	P04075	Fructose-bisphosphate aldolase A	−0.19	26.8	1563
CHORDC1	Q9UHD1	Cysteine and histidine-rich domain-containing protein 1	−0.18	24.87	80
H2AC20	Q16777	Histone H2A type 2-C	−0.18	27.16	2172
PSMD2	Q13200	26S proteasome non-ATPase regulatory subunit 2	−0.17	20.07	163
RPL24	P83731	60S ribosomal protein L24	−0.17	25.7	655
PSMB6	P28072	Proteasome subunit beta type-6	0.15	18.78	139
HMGB2	P26583	High mobility group protein B2	0.16	14.81	63
ANXA3	P12429	Annexin A3	0.17	21.92	1839
NASP	P49321	Nuclear autoantigenic sperm protein	0.18	29.26	109
TCEA1	P23193	Transcription elongation factor A protein 1	0.18	28.89	265
GAR1	Q9NY12	H/ACA ribonucleoprotein complex subunit 1	0.18	28.69	50
C11orf68	Q9H3H3	UPF0696 protein C11orf68	0.21	5.45	30
ABHD10	Q9NUJ1	Palmitoyl-protein thioesterase ABHD10, mitochondrial	0.21	9.92	126
PDHA1	P08559	Pyruvate dehydrogenase E1 component subunit alpha, somatic form, mitochondrial	0.21	20.29	123
BAIAP2	Q9UQB8	Brain-specific angiogenesis inhibitor 1-associated protein 2	0.21	20.28	54
GNB4	Q9HAV0	Guanine nucleotide-binding protein subunit beta-4	0.23	15.84	151
MTHFD2	P13995	Bifunctional methylenetetrahydrofolate dehydrogenase/cyclohydrolase, mitochondrial	0.24	11.46	36
H1-10	Q92522	Histone H1.10	0.24	26.1	572
UQCRQ	O14949	Cytochrome b-c1 complex subunit 8	0.25	26.95	72
TOR1AIP1	Q5JTV8	Torsin-1A-interacting protein 1	0.27	11.54	145
EBP	Q15125	3-beta-hydroxysteroid-Delta(8), Delta(7)-isomerase	0.27	15.2	25
KHSRP	Q92945	Far upstream element-binding protein 2	0.27	27.63	66
TTC38	Q5R3I4	Tetratricopeptide repeat protein 38	0.28	12.45	229
GGH	Q92820	Gamma-glutamyl hydrolase	0.28	7.35	28
HIBADH	P31937	3-hydroxyisobutyrate dehydrogenase, mitochondrial	0.29	19.54	55
GTF2F1	P35269	General transcription factor IIF subunit 1	0.3	22.89	31
RCN3	Q96D15	Reticulocalbin-3	0.32	27.32	21
PPT1	P50897	Palmitoyl-protein thioesterase 1	0.34	7.42	19
EPS8L2	Q9H6S3	Epidermal growth factor receptor kinase substrate 8-like protein 2	0.35	12.88	36
MTX2	O75431	Metaxin-2	0.35	22.57	57
HSPBP1	Q9NZL4	Hsp70-binding protein 1	0.37	27.69	25
SDHB	P21912	Succinate dehydrogenase [ubiquinone] iron-sulfur subunit, mitochondrial	0.38	5.96	68
HEXB	P07686	Beta-hexosaminidase subunit beta	0.39	15.84	128
CETN3	O15182	Centrin-3	0.39	0	17
SERPINE1	P05121	Plasminogen activator inhibitor 1	0.42	3.09	17
PEF1	Q9UBV8	Peflin	0.43	0	16
MIF	P14174	Macrophage migration inhibitory factor	0.45	13.31	106
SCPEP1	Q9HB40	Retinoid-inducible serine carboxypeptidase	0.46	19.98	58
PDCD6	O75340	Programmed cell death protein 6	0.48	12.34	53
CTSZ	Q9UBR2	Cathepsin Z	0.52	21.44	49
ALB	P02768	Albumin	0.53	10.22	97
RPL7	P18124	60S ribosomal protein L7	0.54	2.61	61
SLC7A5	Q01650	Large neutral amino acids transporter small subunit 1	0.57	18.43	45
TUFM	P49411	Elongation factor Tu, mitochondrial	0.66	0	20
SORD	Q00796	Sorbitol dehydrogenase	0.74	0	20
ARF5	P84085	ADP-ribosylation factor 5	1.09	0	714

## Data Availability

The original contributions presented in this study are included in the article/Supplementary Materials. Further inquiries can be directed to the corresponding author(s).

## Supplementary Materials

The following supporting information can be downloaded at: https://www.mdpi.com/article/10.3390/pharmaceutics18080919/s1, Figure S1. (A) TEM micrographs of Ag@MSN-Tf-SeNPs and (B) EDS analysis of the nanosystem; Table S1. Lipids quantified in MDA-MB-231 cells treated with Ag@MSN-Tf-SeNPs at 6h, 24h, 48h, 72h compared with controls (0h) by shotgun lipidomics and normalized by the protein content obtained by the BCA assay.
