# Supplementary material for: Systems-Level Multi-Omics Analysis Resolves the Mechanism of Action of a Novel Multifunctional Nanosystem Against Triple-Negative Breast Cancer
Source: Pharmaceutics. 2026 Jul 27;18(8):919. doi: 10.3390/pharmaceutics18080919 (PMC13516982; doi:10.3390/pharmaceutics18080919)
Supplement: Supplementary file 1 [file pharmaceutics-18-00919-s001.zip › pharmaceutics-4439336-supplementary.pdf]

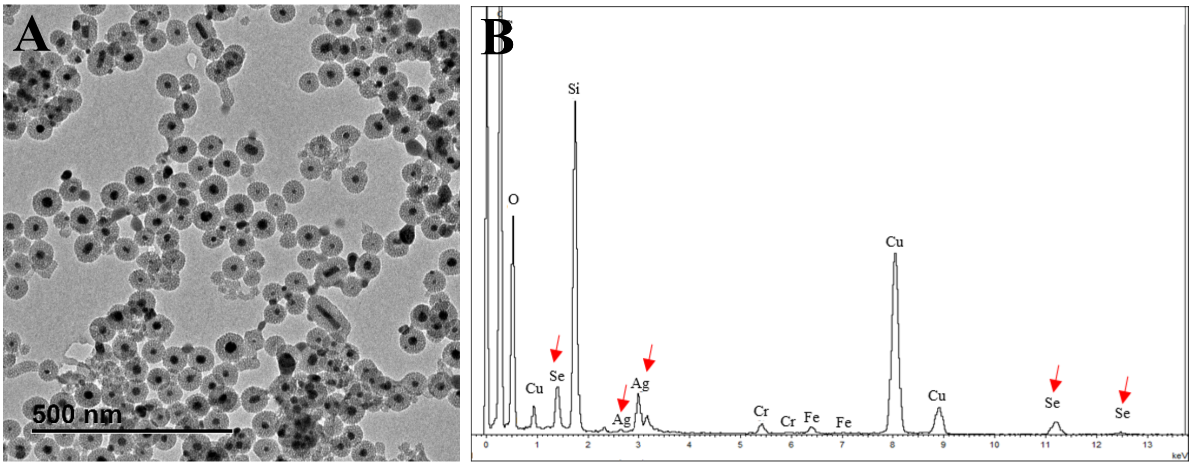

Supplementary Figure S1. (A) TEM micrographs of Ag@MSN-Tf-SeNPs and (B) EDS analysis of the nanosystem.

Table S1. Lipids quantified in MDA-MB-231 cells treated with Ag@MSN-Tf-SeNPs at 6h, 24h, 48h, 72h compared with controls (0h) by shotgun lipidomics and normalized by the protein content obtained by the BCA assay.

	**0h - Controls (pmol/µg protein)**	**6h (pmol/µg protein)**	**24h (pmol/µg protein)**	**48h (pmol/µg protein)**	**72h (pmol/µg protein)**
**CE**	$2,96\cdot10^{-3}$	$3,87\cdot10^{-3}$	$3,62\cdot10^{-3}$	$3,43\cdot10^{-3}$	$3,40\cdot10^{-3}$
**FC**	$1,26\cdot10^{-2}$	$1,20\cdot10^{-2}$	$1,24\cdot10^{-2}$	$1,43\cdot10^{-2}$	$1,56\cdot10^{-2}$
**CL**	$3,32\cdot10^{-3}$	$3,15\cdot10^{-3}$	$2,94\cdot10^{-3}$	$3,52\cdot10^{-3}$	$3,09\cdot10^{-3}$
**LPA**	$3,86\cdot10^{-6}$	$3,41\cdot10^{-6}$	$1,62\cdot10^{-6}$	$7,40\cdot10^{-6}$	$1,45\cdot10^{-5}$
**LPC**	$4,38\cdot10^{-4}$	$3,19\cdot10^{-4}$	$3,62\cdot10^{-4}$	$7,98\cdot10^{-4}$	$7,82\cdot10^{-4}$
**LPE**	$4,65\cdot10^{-4}$	$4,24\cdot10^{-4}$	$4,36\cdot10^{-4}$	$8,55\cdot10^{-4}$	$5,96\cdot10^{-4}$
**LPG**	$7,03\cdot10^{-6}$	$7,82\cdot10^{-6}$	$1,18\cdot10^{-5}$	$1,34\cdot10^{-5}$	$1,29\cdot10^{-5}$
**LPI**	$4,13\cdot10^{-5}$	$2,30\cdot10^{-5}$	$2,09\cdot10^{-5}$	$5,84\cdot10^{-5}$	$1,11\cdot10^{-4}$
**LPS**	$1,08\cdot10^{-5}$	$7,93\cdot10^{-6}$	$8,36\cdot10^{-6}$	$2,37\cdot10^{-5}$	$1,33\cdot10^{-5}$
**PA**	$2,46\cdot10^{-4}$	$1,74\cdot10^{-4}$	$1,49\cdot10^{-4}$	$2,06\cdot10^{-4}$	$4,52\cdot10^{-4}$
**PC**	$3,74\cdot10^{-2}$	$3,56\cdot10^{-2}$	$3,43\cdot10^{-2}$	$3,74\cdot10^{-2}$	$3,65\cdot10^{-2}$
**PE**	$5,73\cdot10^{-3}$	$5,60\cdot10^{-3}$	$5,71\cdot10^{-3}$	$5,63\cdot10^{-3}$	$4,85\cdot10^{-3}$
**PG**	$6,19\cdot10^{-4}$	$6,03\cdot10^{-4}$	$4,96\cdot10^{-4}$	$5,37\cdot10^{-4}$	$5,32\cdot10^{-4}$
**PI**	$4,21\cdot10^{-3}$	$3,98\cdot10^{-3}$	$4,35\cdot10^{-3}$	$4,64\cdot10^{-3}$	$6,22\cdot10^{-3}$
**PS**	$2,21\cdot10^{-3}$	$1,95\cdot10^{-3}$	$1,87\cdot10^{-3}$	$2,27\cdot10^{-3}$	$2,40\cdot10^{-3}$
**Cer**	$1,82\cdot10^{-4}$	$2,42\cdot10^{-4}$	$1,92\cdot10^{-4}$	$3,80\cdot10^{-4}$	$4,28\cdot10^{-4}$
**DAG**	$1,54\cdot10^{-3}$	$1,31\cdot10^{-3}$	$1,21\cdot10^{-3}$	$1,71\cdot10^{-3}$	$2,46\cdot10^{-3}$
**TAG**	$8,36\cdot10^{-3}$	$1,22\cdot10^{-2}$	$1,44\cdot10^{-2}$	$6,42\cdot10^{-3}$	$4,76\cdot10^{-3}$
